# Apropos Data Sharing: Abandon the Distrust and Embrace the Opportunity

**DOI:** 10.1089/dna.2021.0501

**Published:** 2022-01-12

**Authors:** Giorgia Brambilla Pisoni, Mariarosaria Taddeo

**Affiliations:** ^1^ University of London, London School of Hygiene and Tropical Medicine, London, United Kingdom.; ^2^ Oxford Internet Institute, University of Oxford, Oxford, United Kingdom.; ^3^ Alan Turing Institute, London, United Kingdom.

**Keywords:** data sharing, digital ethics, European Union, human genomics, privacy, rights, public health

## Abstract

In this commentary, we focus on the ethical challenges of data sharing and its potential in supporting biomedical research. Taking human genomics (HG) and European governance for sharing genomic data as a case study, we consider how to balance competing rights and interests—balancing protection of the privacy of data subjects and data security, with scientific progress and the need to promote public health. This is of particular relevancy in light of the current pandemic, which stresses the urgent need for international collaborations to promote health for all. We draw from existing ethical codes for data sharing in HG to offer recommendations as to how to protect rights while fostering scientific research and open science.


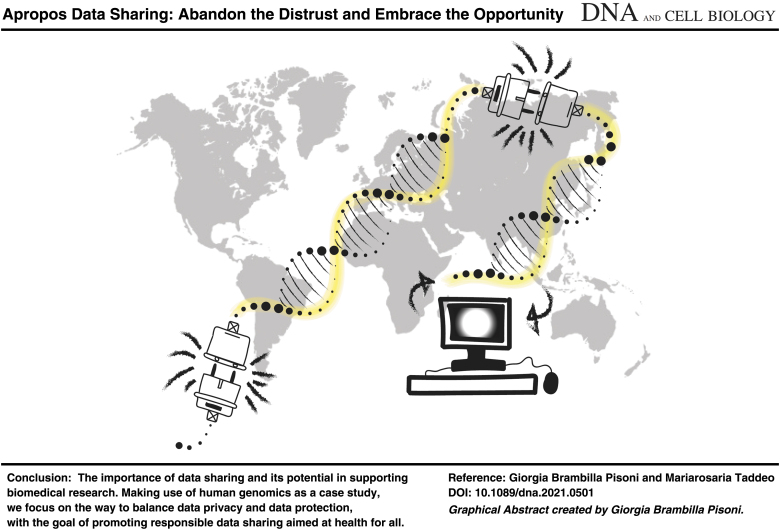


## Introduction

### Human genomics as the health science of big data

Human genomics (HG) is the research field focusing on the analysis of DNA sequences and of their mutations across individuals and populations, with the goal of identifying strong correlations between the information contained in the DNA and specific disease profiles. HG research aims at uncovering disorder patterns caused by genetic factors, anticipating their onset, and enabling preventive interventions. HG paves the way to personalized and predictive medicine (Roth, 2019), to a much better understanding of human pathophysiology, and to developing more intervention options for diseases (Berens and Marchant, 2004; Goodwin *et al.*, 2016).

The basic idea is that the more data that can be gathered and analyzed, the more comprehensive will be the depth of our understanding of the genetic determinants of disease. Indeed, the use of large data sets and comparison between different ones gives higher statistical confidence to emerging patterns. Large data sets also facilitate the identification of low frequency events, associated for instance with the onset of rare diseases, whose detection would be unlikely otherwise (Francis, 2014).

Large volumes of data pose the need for huge platforms in which to store and analyze the data and metadata originating from study samples. According to OmicsMaps (in disuse at present), >2500 high-throughput next-generation sequencing instruments were in use across >60 countries in 2015. Estimates from 2015 predicted that, if run at full capacity, these instruments had the power to generate many zettabases (i.e., trillions of billions of sequenced bases) of data by 2025, and to sequence up to 2 billion human genomes by 2027 (Stephens *et al.*, 2015; The Medical Futurist, 2018). More recently, HG projects around the world and their metadata have been collected in the Genomes OnLine Database (GOLD). Its current version includes >1.17 million entries and contains >600 metadata fields. Considering that a single human genome (in the form of a compressed file, composed of about 30 sampling rounds for proper information quality) requires 100 gigabytes for proper storage, data storage capacity will need to scale up dramatically as genome sequencing keeps increasing (Fleishman, 2015; Stephens *et al.*, 2015; Mukherjee *et al.*, 2021).

Once collected and stored properly, data need to be shared among scientists to support research. Both storing and sharing the data pose serious ethical risks, which, if left unmitigated, may hamper the development of HG and its many benefits for human health.

### Ethical risks of the management of human genomic data

One of the main risks of collecting and sharing HG data is the retrieval of individual identities associated with the samples, and the consequent threats that this may represent in terms of discrimination and stigmatization of individuals, or of minority/vulnerable groups (Mailman *et al.*, 2007; Provost and Fawcett, 2013; Kosseim *et al.*, 2014; Byrd *et al.*, 2020). To mitigate these risks, protocols have been defined for sharing and accessing data securely, including the one outlined by the National Center for Biotechnology Information (NCBI) in 2007, the so-called “upon-request sharing” protocol (Mailman *et al.*, 2007). This protocol is used when data are shared within a close group of participants, often collaborating on the same project (Byrd *et al.*, 2020).

Each form of data sharing is tightly regulated by specific policies that, despite aiming at encouraging the acceleration of discovery, have a strong focus on privacy protection. The risk here is that too severe measures curtailing data sharing may hinder progress in HG and related progress for public health, particularly when they encroach on cross-border sharing of data. As stressed by Molnár-Gábor and Korbel:

“Data sharing across borders has been transformative for research on both rare diseases and cancer. Each individual rare disease is so scarce that individual centers and often entire countries may lack the patient cohorts to meaningfully interpret the disease. […] One example is research on childhood medulloblastoma, where cross-border sharing of patient genetic and clinical data within Europe and beyond led to breakthroughs that uncovered the frequent hereditary basis of the disease and led to new recommendations for clinical management,” (Molnár-Gábor and Korbel, 2020).

In the following sections, we focus specifically on the governance measures defined for HG data in the European Union (EU) to examine the friction between the EU governance for protection of personal data and fostering progress in HG.

#### EU governance of genomic data

The personal data of EU citizens collected as part of HG research are regulated according to the General Data Protection Regulation (GDPR). The GDPR offers one of the most advanced and comprehensive directives for the protection of personal data to date. However, its heterogeneous implementation across EU Member States—together with some of its strict provisions with respect to access and sharing of personal data (particularly health data)—have proven to be problematic when dealing with data collection and sharing for research purposes. This is even more the case for HG research.

A 2020 report by the Public Health Genomics (PHG) Foundation, for example, indicates that the application of the GDPR directive to genomic data poses significant challenges with respect to:

- “Uncertainty in determining when the GDPR applies to collaborators in genomic initiatives, in particular when professionals may become ‘joint controllers' and when those outside the EU must comply with the GDPR;
- Uncertainty in determining when genetic, genomic and health-associated data are *de facto* ‘personal data’ governed by the GDPR and whether data that have been de-identified (e.g., through pseudonymisation) remain personal data;
- Meeting the requirements for a lawful basis for processing personal data and specific conditions for processing ‘special category’ (e.g., health or genetic) data;
- Fulfilling data subject rights and meeting obligations under the GDPR and DPA 2018; and
- Making data accessible to others or data sharing both within the EU/EEA and to ‘third countries’” (Mitchell *et al.*, 2020, p. 5).

HG scientists have called for an ethical code of conduct to address the uncertainties identified in the PHG Foundation report, and to guide scientists in defining ethically sound trade-offs between the protection of individual rights and the progress of scientific research and public health interventions based on open science and precision medicine (Molnár-Gábor and Korbel, 2020). We agree with this view. We believe that, as for other domains that have been transformed by digital technologies, an ethical approach is essential to leverage the potential of digital innovation to improve science and public health and to address ethical risks before these lead to social rejection and too strict regulation, which would eventually hamper scientific progress (Floridi and Taddeo, 2016; Morley *et al.*, 2020).

#### European codes of conduct for data sharing, lessons learned, and the path ahead

The value of data for research, and in particular biomedical research, is recognized in the EU strategy for data governance (Roberts *et al.*, Forthcoming). Notably, the European Commission aims to adopt a communication

“supporting data infrastructure to advance research, diseases prevention and personalised health and care in key areas including rare, infectious and complex diseases” (European Commission, n.d.).

Maximizing the research potential of data requires two elements: normative and technological. On the normative front, best practices and codes of conduct guiding practitioners to make ethically sound decisions are essential. The GDPR (Art. 40) envisages the development of these codes. Indeed, there are initiatives focusing on developing such codes for the biomedical research—for example, BBMRI-ERIC, a European biobanking research infrastructure, announced in 2017 that it would develop an EU-wide “Code of Conduct on Health-Related Data” (Nicholson, 2017). Scientists working in HG have also identified a number of key principles—for example, broad consent, sharing of finding with data subjects, portability, access, withdrawal, and complete disclosure—that should be central for these codes (Phillips *et al.*, 2020).

Ethical codes of conduct for HG do not need to be defined from scratch. These should draw from, and be consistent with, research and medical ethics codes (which have already adopted by universities and research institutions globally), with the focus being on fundamental principles, such as privacy protection of data subjects, their autonomy, consent, and withdrawal. HG ethical codes of conduct should not substitute for laws; rather they offer postcompliance guidance and indicate what ought to be done or not to be done,

“*over and above* the existing regulation, not against it, or despite its scope, or to change it, or to by-pass it (e.g., in terms of self-regulation)” (Floridi, 2018, p. 4).

Ethical implications of HG also stem from the technological infrastructure that supports this research. Given the volume of data involved, genomics will increasingly rely on cloud infrastructures to store, share, and analyze data. Past mistakes have shown that it is crucial to ensure that the cloud services that underpin this research not only respect fundamental rights of data subjects (such as privacy and anonymity) but also that they guarantee security of the data, and access to legitimate users, portability, while ensuring redundancy. An ethical code of practice for HG should, therefore, extend its focus to include ethical requirements for the computational infrastructures underpinning HG research, to ensure data control and security, confidentiality, accountability, and redundancy. Nascent EU standards for cloud computing as developed by GAIA-X point in the right direction (GAIA-X, 2020).

Ethical codes of practice for HG should reconcile the normative and technological element while harmonizing the protection of data subjects with scientific progress. We offer three recommendations that may facilitate the achievement of these goals.

##### Field-wide code of conduct and third-party oversight board

Codes of conduct do not work if not embraced by institutions and professional communities. An HG ethical code of conduct should be embraced by the research field. For example, adherence to a field-recognized code of conduct should be a requirement for funding allocation and publication. A third-party oversight board would be ideally placed to update the code of practice as science and technology develop, to monitor the adoption of the code, and to offer guidance when considering cross-border access to data between different ethical and cultural contexts, and when researchers may face particularly problematic trade-offs.

##### Group privacy

“Privacy as a group right is a right held by a group as a group rather than by its members severally. It is the group, not its members, that is correctly identified as the right-holder. A typical example is the right of self-determination, which is held by a nation as a whole” (Floridi, 2014, p. 1). The protection of group privacy is crucial in the age of big data and artificial intelligence, where data collection often leads to identify categories, that is, *groups* of individuals rather than to single out a specific person. This is why it is important that these codes include explicit measures to protect the rights and ensure fair treatment of any groups that are identified by HG research (Morley *et al.*, 2020; Taddeo, 2020).

##### Digital sovereignty

EU initiatives for the governance of the digital are increasingly centered around the concept of digital sovereignty (Roberts *et al.*, 2021). When considering the topic of this commentary, this concept has two implications. The first is purely normative: managing the data of EU citizens according to the fundamental values of the EU. These values need to be respected independently of the location in which data are stored and analyzed. This is already mandated by the GDPR and should be made explicit in any code of conduct for HG. The second implication is technical and has a strong focus on control of access to the data. Digital sovereignty calls for ensuring that the genomic data of EU citizens are stored in data centers located within EU borders. This is not a measure to limit legitimate cross-border sharing. Maintaining data on EU territory does not imply that data cannot be shared outside the EU borders. However, the physical location of data within EU borders reduces the chances that the personal sensitive data of EU citizens is accessed by other governments, unauthorized parties, and risk to be treated in ways that do not respect EU values and laws (Mildebrath, 2020).

## Conclusions and Future Perspectives

Digital technologies hold great promise for social good, and data for HG research are no exception. This promise is solid, but it will not materialize without adequate ethical governance to help bring it about in ways that are coherent with the fundamental values of our societies, and that will ensure that scientific progress does not come at the expense of individual and group rights (Floridi *et al.*, 2020).

The EU is a global leader in digital governance and should also lead the debate on the ethical governance of data in science. HG is a great benchmark to this end. Defining an international field-wide code of conduct for data collection, managing, and sharing data in HG would have important positive implications. For example, it would avoid digital ethics dumping, that is

“the malpractice of (a) exporting research activities about digital processes, products, services, or other solutions, in other contexts or places (e.g., by European organizations outside the EU) in ways that would be ethically unacceptable in the context or place of origin and (b) importing the outcomes of such unethical research activities” (Floridi, 2019, p. 190).

In an increased globalized world, the benefits of HG research should be accessible globally to everyone and become an inclusive tool for personalized health care. For this to happen, HG must be developed on a global scale—thus, an international code of conduct is key to extend research in genomics outside the boundaries of high-income countries, without encroaching upon individual and group rights. In this sense, defining such a code is a key step to improving global health.

However, if not coupled with substantive measures to ensure inclusive representation and access to the results of research on HG, these codes of practice risk to deepen the divide between those able to access state-of-the-art health care and those who do not (Hilton *et al.*, 2010; Cohn *et al.*, 2017; Landry *et al.*, 2018). To this end, codes of practice for research on HG should go beyond data management and include measures to foster representativeness of data and access to the research results following the principle of distributive justice.

The definition of these measures is outside the scope of this opinion article, but we wish to conclude it by remarking that without a strong focus on representativeness of databases and equal access to the results of research on HG, principles to develop ethically sound collection, storage, and access of HG data are bound to offer sterile guidance for a research that has the potential to improve human health at global scale.
